# A Decade of Progress in Wearable Sensors for Fall Detection (2015–2024): A Network-Based Visualization Review

**DOI:** 10.3390/s25072205

**Published:** 2025-03-31

**Authors:** Yifei Li, Pei Liu, Yan Fang, Xiangyuan Wu, Yewei Xie, Zhongzhi Xu, Hao Ren, Fengshi Jing

**Affiliations:** 1 Hikvision Research Institute, Hangzhou 310051, China; liyifeisn@zju.edu.cn (Y.L.); liupei9@hikvision.com (P.L.); 2 College of Civil Engineering and Architecture, Zhejiang University, Hangzhou 310027, China; 3 Faculty of Data Science, City University of Macau, Taipa, Macao SAR 999078, China; d24091110131@cityu.edu.mo (Y.F.); d23091110031@cityu.edu.mo (X.W.); 4 Programme in Health Services and Systems Research, Duke-NUS Medical School, Singapore 169857, Singapore; yewei.xie@u.duke.nus.edu; 5 School of Public Health, Sun Yat-sen University, Guangzhou 510080, China; xuzhzh26@mail.sysu.edu.cn; 6 Guangzhou Key Laboratory of Smart Home Ward and Health Sensing, The Affiliated Guangdong Second Provincial General Hospital of Jinan University, Guangzhou 510317, China; 7 UNC Project-China, The University of North Carolina at Chapel Hill, Chapel Hill, NC 27599, USA

**Keywords:** wearable sensor, Internet of Things, fall detection, fall prevention, inertial sensor, pre-impact fall

## Abstract

Over the past decade, wearable sensors for fall detection have gained significant attention due to their potential in improving the safety of elderly users and reducing fall-related injuries. This review employs a network-based visualization approach to analyze research trends, key technologies, and collaborative networks. Using studies from SCI- and SSCI-indexed journals from 2015 to 2024, we analyzed 582 articles and 65 reviews with CiteSpace, revealing a significant rise in research on wearable sensors for fall detection. Additionally, we reviewed various datasets and machine learning techniques, from traditional methods to advanced deep learning frameworks, which demonstrate high accuracies, F1 scores, sensitivities, and specificities in controlled settings. This review provides a comprehensive overview of the progress and emerging trends, offering a foundation for future advancements in wearable fall detection systems.

## 1. Introduction

Falls are a major public health concern, particularly among older adults, and often lead to debilitating injuries, a loss of independence, and a significant increase in healthcare expenditures. As the global population continues to age, the incidence of falls and their associated risks is expected to rise, making the development of effective fall detection systems an urgent priority. Falls not only have a profound impact on the health and well-being of older individuals but also pose a significant burden on healthcare systems, resulting in prolonged hospitalization and rehabilitation times and increased care needs. Given the aging demographic and rising healthcare costs, addressing this issue through innovative technological solutions is critical [1].

In recent years, wearable sensors have emerged as a promising solution for fall detection due to their portability, affordability, and ability to continuously monitor human movement in real time [2,3]. These sensors are being increasingly integrated into wearable devices, such as wristbands, smartwatches, and body-mounted units, and are often equipped with advanced components such as accelerometers, gyroscopes, and pressure sensors [4]. For example, Ahmet et al. [5] developed a fall detection system employing triaxial accelerometers and gyroscopes, while Chan et al. [6] integrated pressure sensors with machine learning algorithms to enhance detection accuracy. Similarly, Muheidat et al. [7] showcased a wearable device that successfully utilized real-time data processing to promptly alert caregivers after a fall. Such studies highlight the potential of wearable sensor technologies to not only achieve high detection accuracy but also enable early intervention, thereby reducing the risk of further injury [8].

The rapid development and application of wearable sensors for fall detection over the past decade have garnered significant attention from researchers, clinicians, and technologists. Numerous studies have explored various sensor technologies, algorithms, and system designs to improve the accuracy and reliability of fall detection devices. Moreover, these advancements have led to the integration of wearable sensors with other technologies, such as the Internet of Things (IoT) and artificial intelligence (AI), further enhancing their functionality and potential for personalized fall detection solutions [9,10]. As this field continues to evolve, it is essential to assess the current state of research and identify emerging trends, challenges, and opportunities for future innovation [9].

Building on these advancements, this study presents a bibliometric analysis of 582 publications over the past decade that were sourced from the Web of Science (Core Collection). By mapping key researchers, institutions, and emerging research trends, our analysis aims to provide a comprehensive overview of the progress made in wearable sensor-based fall detection. This work intends to inform and guide future research and development efforts, assisting researchers, developers, and policymakers in advancing the design and implementation of more effective fall detection systems [11,12].

## 2. Materials and Methods

**Figure 1:**
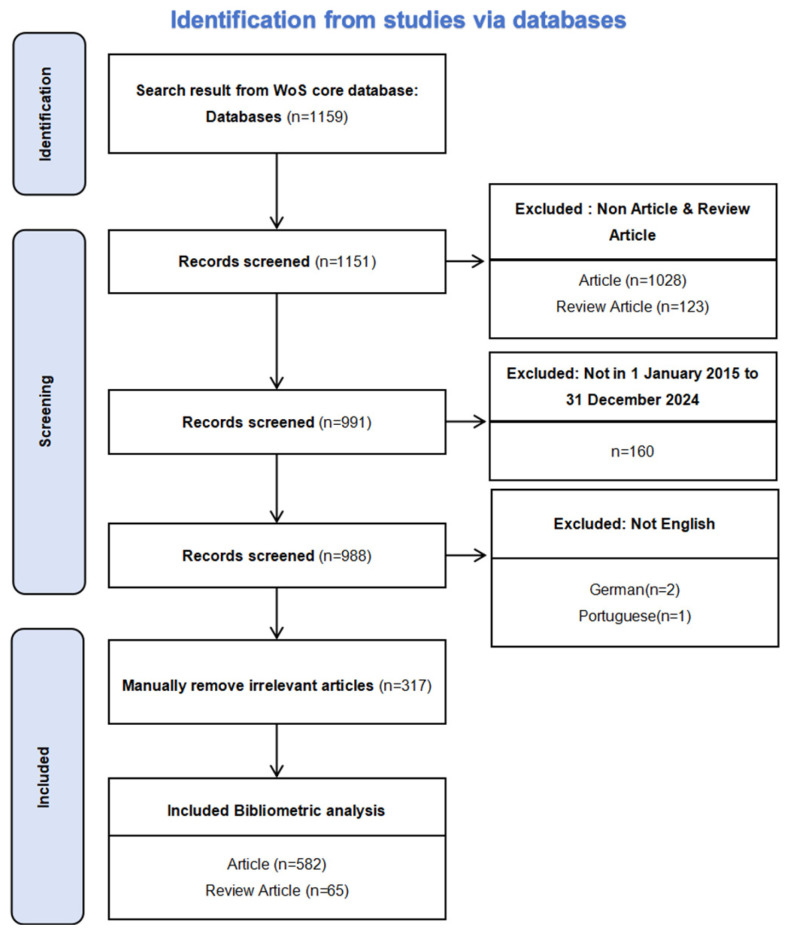
Flowchart of the search strategy in the study.

Following the methodology proposed by Donthu [13], this bibliometric analysis was conducted in five steps. First, the objectives and scope of the study were defined, with a specific focus on wearable sensor technologies for fall detection. The second step involved filtering out non-English articles and publications that were not research or review articles. In the third step, articles that were not directly related to the research topic were manually excluded based on their relevance to wearable sensors for fall detection. The flowchart of the research strategies for this study is presented in Figure 1.

### 2.1. Data Sources

This study utilized the Web of Science (Core Collection) database as the primary data source. The Web of Science, which is managed by Clarivate Analytics, is a highly regarded digital research database; it is widely acknowledged for its comprehensive and reliable coverage as it encompasses a broad spectrum of publications across various fields. It is considered one of the most suitable databases for bibliometric analysis [14]. Additionally, we also searched Scopus and PubMed, and after filtering for SCI/SSCI articles, we found that all such articles were included in the Web of Science search results.

### 2.2. Search Strategy

Data were collected from 1 January 2015, to 31 December 2024, and to maintain consistency, no updated articles were included from this period. The following search terms were used for the screening processes:

(TS = (“Wearable” OR “Sensor” OR “Internet of Everything” OR “IoT” OR “Internet of Things” OR “Artificial Intelligence of Things” OR “AIoT”) AND (“Fall Detection” OR “Fall Recognition” OR “Fall Monitoring” OR “Fall Prediction” OR “Fall Prevention”)).

This search yielded 1159 records from the SCI and SSCI indexes. After the exclusion of publications that were not research or review articles and those not in English, 988 records were used for further analysis.

### 2.3. Data Analysis and Visualization

An initial analysis of the publication counts, citation numbers, and global trends was conducted using the Web of Science’s search and citation tools. Subsequently, the data were exported in a “plain text format” as “full records and cited references” for further analysis using CiteSpace (6.3.R1 (64-bit) Basic, developed by Drexel University, Prof. Chaomei Chen; Address of Drexel University: 3141 Chestnut Street, Philadelphia, PA 19104, USA), a bibliometric tool designed to find research trends and knowledge structures [15]. CiteSpace uses citation analyses to identify key publications, research hotspots, and influential authors and generate time-based visual maps to illustrate the evolution of a research field [16]. These visualizations, which present the structure, patterns, and distribution of scientific knowledge, are often referred to as “scientific knowledge maps” [17].

## 3. Results

### 3.1. Global Trends in Publications and Citations

**Figure 2:**
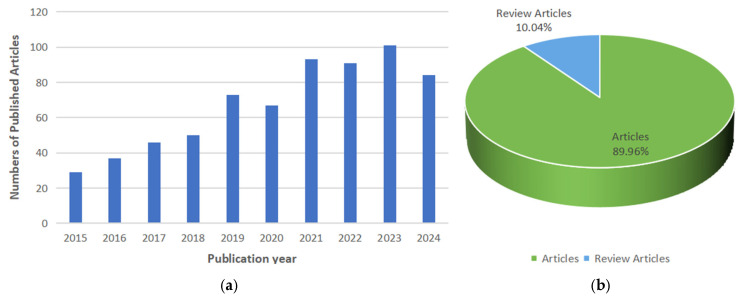
(**a**) Publication trends in wearable sensors for fall detection. (**b**) Distribution of document types between articles and reviews.

Between 1 January 2015, and 31 December 2024, 647 articles focusing on the application of wearable sensors for fall detection were retrieved from the SCI and SSCI indexes. Of these, 582 were research articles and 65 were review articles. From 2019 to 2024, the annual publication count did not exceed 60 (Figure 2a). However, a notable increase occurred in 2021 and 2022, with publications surpassing 90 in each of these years. The year 2023 saw the highest publication count of the decade, exceeding 100 papers. Figure 2b demonstrates that 89.96% of the documents are research articles, while 10.04% are review articles, suggesting a predominant focus on primary research contributions, with a smaller proportion dedicated to synthesizing existing knowledge.

### 3.2. Analysis of the Distribution of Published Articles by Country and Region

**Figure 3:**
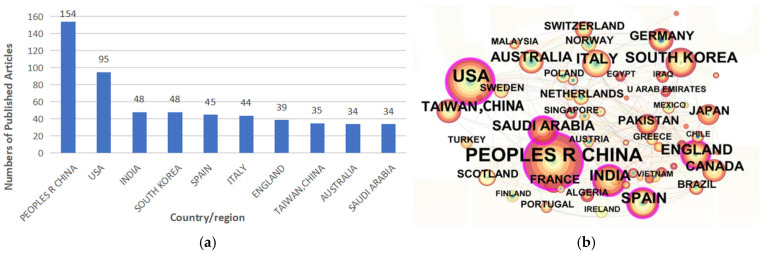
Analysis of the distribution of published articles by country/region in terms of global research productivity and influence in wearable sensors for fall detection: (**a**) top 10 productive countries/regions; (**b**) network visualization map.

Publication data by country/region (Figure 3a) reveal that China leads with 154 publications, followed by the United States with 95 publications; India and South Korea are tied for third place, each with 48 publications. Among the top ten countries/regions by publication count, six have published more than 40 papers (Table 1). The map consists of 67 nodes, 314 connections, a density of 0.142, a modularity of Q = 0.4643, and a Weighted Mean Silhouette of S = 0.7408, indicating close and robust international collaboration networks (Figure 3b), with strong relationships between both developed and developing countries/regions such as China, the United States, South Korea, and India.

### 3.3. Analysis of the Distribution of Published Articles by Institution

**Figure 4:**
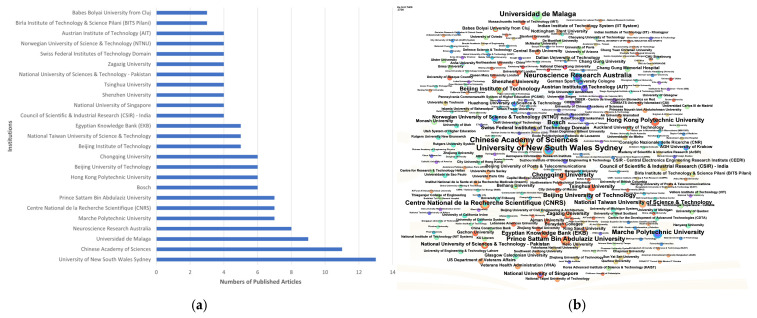
Analysis of the distribution of published articles by institution in terms of global research productivity and influence in wearable sensors for fall detection: (**a**) top 25 productive institutions; (**b**) network visualization map of the institutions.

Data from the Web of Science (WoS) show that over 200 institutions (263 in total) have published articles in the field of IoT applications. Figure 4a highlights the top 25 contributing institutions by publication count. The University of New South Wales Sydney, the Chinese Academy of Sciences, and the Universidad de Malaga lead with 13, 11, and 9 publications, respectively. Fourteen institutions have published more than five papers, signaling ongoing advancements in wearable sensors for fall detection. In Figure 4b, the map includes 263 nodes, 263 connections, a density of 0.0076, a modularity of Q = 0.4643, and a Weighted Mean Silhouette of S = 0.7408, depicting the collaboration network among these institutions, with thicker lines indicating stronger collaborative ties.

### 3.4. Analysis of the Distribution of Keywords

**Figure 5:**
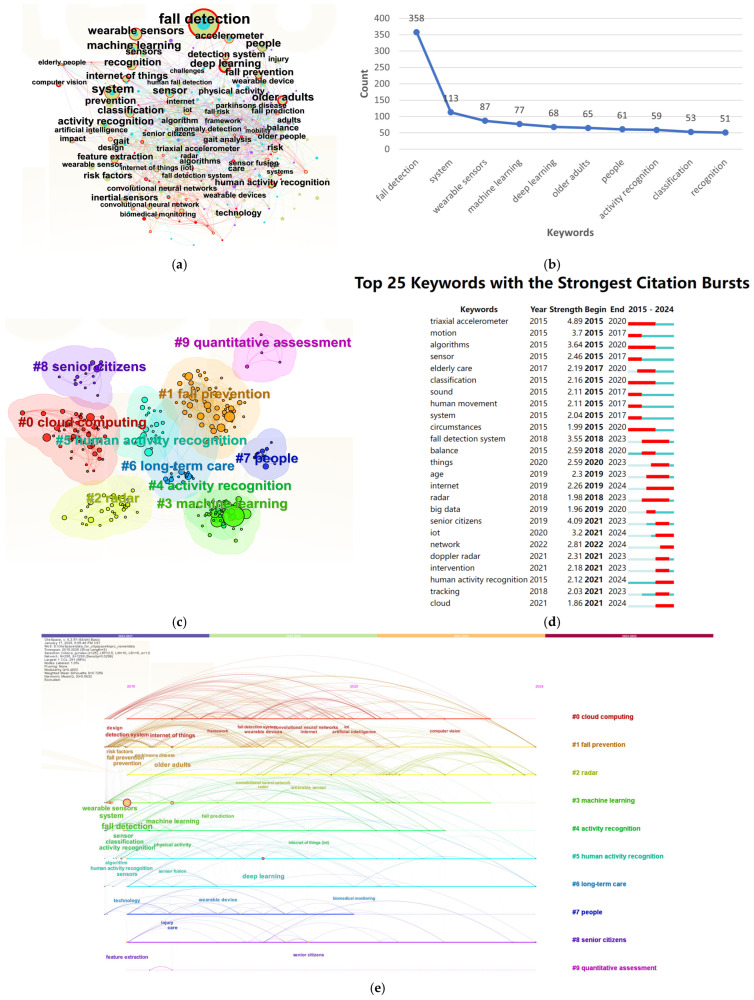
Analysis of the distribution of keywords related to wearable sensors for fall detection: (**a**) network visualization map of keywords; (**b**) top 10 keywords for wearable sensors for fall detection; (**c**) cumulative occurrences of selected keywords from 2015 to 2024; (**d**) top 25 keywords with the strongest citation bursts in academic research (2015–2024); and (**e**) timeline chart for the period of 2015–2024.

A visualization map of keywords related to wearable sensors for fall detection was created using CiteSpace during 2015–2024 with time slices of three years. The map includes 295 nodes and 1293 connections, with a density of 0.0298, a modularity of Q = 0.4643, and a Weighted Mean Silhouette of S = 0.7408. Figure 5a displays the keyword network. The top 10 most frequent keywords include “fall detection” (358 occurrences), “system” (113 occurrences), “wearable sensors” (87 occurrences), “machine learning” (77 occurrences), “deep learning” (68 occurrences), “older adults” (65 occurrences), “people” (61 occurrences), “activity recognition” (59 occurrences), “classification” (53 occurrences), and “recognition” (51 occurrences) (Figure 5b, Table 2). The prevalence of keywords such as “wearable sensors” and “fall detection” underscores the continuous emergence and widespread application of this field.

Figure 5c illustrates the co-occurrence network of keywords, which clusters into ten distinct regions: cloud computing, fall prevention, radar, machine learning, activity recognition, human activity recognition, long-term care, people, senior citizens, and quantitative assessment. Each cluster is numbered, with lower numbers indicating clusters with more keywords. The clustering analysis yielded a Q-value of 0.4602 and an S-value of 0.7256, indicating significant clustering results, thus confirming the reliability of the analysis.

The burst chart (Figure 5d) identifies 25 keywords with significant citation bursts from 2015 to 2024, highlighting periods of increased attention driven by research trends or technological advancements. Notably, “triaxial accelerometer” experienced a burst in 2015, with a burst intensity of 4.89. This term, referring to a device that measures acceleration along three axes, reflects the growing interest in wearable devices for human activity monitoring. Similarly, “fall detection system” surged in 2018, and “internet” exhibited a burst between 2019 and 2024, indicating an increasing integration of the Internet of Things (IoT) with fall detection.

Finally, Figure 5e presents a timeline map that tracks the evolution of key research topics in the field. The intertwining of nodes and lines illustrates the interdisciplinary connections between the IoT, wearable devices, human activity recognition, and fall detection. In particular, keywords such as “wearable sensors”, “human movement”, “fall detection system”, and “human activity recognition” represent crucial research areas, as their prominence grew over time, especially between 2021 and 2024.

### 3.5. Fall Detection Dataset

The availability of diverse and comprehensive datasets plays a crucial role in advancing the development of fall detection systems. A variety of datasets have been created for this purpose, often employing different sensor modalities, such as accelerometers, gyroscopes, thermal cameras, and audio signals, to capture and categorize human activities, including falls. These datasets facilitate the training and validation of machine learning models, which are essential for improving the accuracy and robustness of fall detection algorithms. Table 3 provides an overview of the most widely used fall detection datasets, detailing the type of data, the number of samples, and their respective characteristics.

One prominent dataset is Fall Detection [18] from Kaggle, which consists of 374 training images and 111 validation images categorized into three human activity types: falling, walking, and sitting. This dataset is useful for image-based fall detection models, although its relatively small size may limit its generalizability.

The Elderly Fall Prediction and Detection [19] dataset, also available on Kaggle, includes 2040 parameter records focused on predicting and detecting falls in older individuals. This dataset is valuable for research focused on fall prediction in a specific population, but it lacks multi-modal data integration, which could enhance the overall accuracy of these models.

Another dataset, Fall vs. Normal Activities [20], contains 96,801 dimensional values collected using accelerometers and gyroscopes to differentiate between falls and normal activities based on the number of signal dimensions. While the large volume of data makes this dataset highly informative, the focus on accelerometer and gyroscope signals alone may limit its ability to detect falls under different real-world conditions.

The Thermal Mannequin Fall Image dataset [21] innovatively uses thermal imaging for fall detection, with 22 videos taken using a thermal imager and boundary box annotations. Although thermal imaging is advantageous in low-visibility scenarios, this dataset is limited by the use of mannequins rather than human subjects, affecting its real-world applicability.

The SisFall [22] dataset contains 1798 and 2706 data points, respectively, on both falls and activities of daily living (ADLs), which were recorded using a combination of accelerometers and gyroscopes. Although this is a comprehensive dataset, its reliance on only a small group of elderly subjects may not fully capture the diversity of fall scenarios.

Other datasets such as Thermal Fall Detection and Activity [23], SAFE [24], and KFALL [25] expand on the use of thermal and audio signals, further enhancing the range of sensor modalities for fall detection. The SAFE dataset, in particular, uses machine learning and deep learning techniques to analyze sound signals for fall detection, highlighting the potential for sound-based detection systems.

**Table 3:** Available datasets of fall detection.

Dataset	Description	Fall Detection Data Included	Is It Simulated?
Fall Detection [18]	This dataset contains images of three categories of human activities (falling, walking, and sitting).	374 images for training and 111 images for validation.	Yes
	URL: https://www.kaggle.com/datasets/uttejkumarkandagatla/fall-detection-dataset (accessed on 18 February 2025)		
Elderly Fall Prediction and Detection [19]	This is a fall detection and prediction dataset for elders.	2040 parameter records related to fall detection.	Yes
	URL: https://www.kaggle.com/datasets/laavanya/elderly-fall-prediction-and-detection (accessed on 18 February 2025)		
Fall vs. Normal Activities [20]	Based on two signals, the accelerometer and gyroscope, each signal has three dimensions. The fall status is determined by calculating the number of dimensions.	96,801 dimensional values related to the accelerometer and gyroscope.	Yes
	URL: https://www.kaggle.com/datasets/enricogrimaldi/falls-vs-normal-activities (accessed on 18 February 2025)		
Thermal Mannequin Fall Image [21]	The video captured using the Hikvision DS-2TD2235D thermal imager at the Aalborg port in Denmark focuses on frames before, during, and after the model is thrown into the port.	22 videos taken with a thermal imager, extracted images sampled from every 5 frames of the videos, and boundary box annotations in YOLOv5 format.	Yes
	URL: https://www.kaggle.com/datasets/ivannikolov/thermal-mannequin-fall-image-dataset (accessed on 18 February 2025)		
SisFall [22]	A dataset of falls and activities of daily living (ADLs) was acquired with a device composed of two types of accelerometers and one gyroscope.	2706 ADLs and 1798 falls, including data from 15 healthy and independent elderly persons.	Yes
	URL: https://doi.org/10.1109/TETC.2020.3027454 (accessed on 18 February 2025)		
Thermal Fall Detection and Activity [23]	Multilayer preprocessing is applied to the original thermal images.	1600 fall instances and 1600 non-fall instances.	Yes
	URL: https://www.kaggle.com/datasets/boeychunhong/thermal-fall-detection-and-activity-dataset (accessed on 18 February 2025)		
SAFE [24]	ML and deep learning (DL) algorithms are employed for fall detection using sound signals.	950 audio samples and 475 fall events.	Yes
	URL: https://www.sciencedirect.com/science/article/pii/s2352648324000953 (accessed on 18 February 2025)		
KFALL [25]	This is applicable for pre-impact fall detection and post-fall detection.	21 types of ADLs (activities of daily living) and 15 types of falls.	Yes
	URL: https://sites.google.com/view/kfalldataset (accessed on 18 February 2025)		
UP-Fall [26]	This is a multi-modal approach (wearable sensors, environmental sensors, and visual devices).	11 activities, with 3 trials per activity.	Yes
	URL: https://sites.google.com/up.edu.mx/har-up/ (accessed on 18 February 2025)		
UniMiB SHAR [27]	A smartphone with a three-axis gravity accelerometer was used to measure acceleration.	11,771 individual human activity and fall samples from 30 subjects aged between 18 and 60 years.	Yes
	URL: http://www.sal.disco.unimib.it/technologies/unimib-shar/ (accessed on 18 February 2025)		
MobiFall [28]	The inertial sensors of a smartphone (3D accelerometer and gyroscope) were positioned in a trouser pocket.	342 ADLs and 288 falls.	Yes
	URL: https://github.com/yehowlong/MobiAct_Dataset_v2.0-MobiFall_Dataset_v2.0 (accessed on 18 February 2025)		
DLR [29]	IMU	961 ADLs and 56 falls.	Yes
	URL: https://dl.acm.org/doi/abs/10.1145/1864431.1864480 (accessed on 18 February 2025)		
UMAFall [30]	The data come from a smartphone worn in the right thigh pocket and four wearable sensors placed on the ankle, waist, right wrist, and chest.	3 types of falls and 8 ADLs (activities of daily living).	Yes
	URL: https://www.diana.uma.es/?page_id=312 (accessed on 18 February 2025)		
Tfall [31]	Smartphones were carried by the subjects every day to ascertain ADLs.	Continuous ADLs and 240 falls.	Yes
	URL: https://journals.plos.org/plosone/article?id=10.1371/journal.pone.0094811 (accessed on 18 February 2025).		
Vilarinho et al. [32]	This was a combination of a smartphone and a smartwatch.	7 ADL activities and 12 types of falls.	Yes
	URL: https://ieeexplore.ieee.org/abstract/document/7363260 (accessed on 18 February 2025)		
OCCU [33]	This collected occluded falls using two Kinect cameras.	5 subjects experiencing 60 occluded falls and similar/different actions in the first dataset.	Yes
	URL: http://sites.google.com/site/occlusiondataset (accessed on 18 February 2025)		

While these datasets provide a strong foundation for fall detection research, they also present certain limitations. Many datasets are simulated, which may not fully represent the complexities of real-world fall events. Additionally, most datasets tend to be relatively small in scale or focus on specific populations, such as elderly individuals, limiting the generalizability of the models trained on them. Furthermore, the use of limited sensor modalities (e.g., accelerometer and gyroscope data) can restrict the accuracy of fall detection under varied environmental conditions.

To address these challenges, future fall detection datasets could benefit from incorporating a broader range of sensor modalities, including visual, environmental, and multi-modal sensor data. For instance, combining accelerometer and gyroscope data with thermal imaging, audio signals, and even wearable sensors could improve the robustness of fall detection systems, making them more comparable to real-world scenarios. Additionally, datasets that include data from a more diverse set of subjects, such as individuals with different physical abilities, health conditions, and environmental contexts, would create generalizable fall detection systems.

Moreover, increasing the scale of these datasets by gathering more samples from real-world environments could further enhance the performance of fall detection algorithms. Large, diverse, and multi-modal datasets are essential for advancing the field of fall detection, and future research should focus on overcoming these data limitations to create more accurate and reliable systems.

### 3.6. Machine Learning for Fall Detection

Table 4 summarizes a diverse range of datasets and methodologies for fall detection, which primarily include data from elderly populations, but data from children, healthy adults, and patients with specific conditions such as Parkinson’s disease can also be found in these datasets. The studies leverage a variety of data sources—including inertial measurement units, EEG signals, radar, RFID systems, and depth images—to capture fall-related events. Methodologically, the approaches range from traditional machine learning techniques (e.g., support vector machines and k-nearest neighbors) to advanced deep learning frameworks such as convolutional neural networks (CNNs), long short-term memory networks (LSTMs), Transformers, and object detection models like YOLO. Several works incorporate innovative preprocessing strategies, such as mixed-radix FFT and background subtraction, as well as sensor fusion and attention mechanisms, to enhance feature extraction and classification accuracy. Notably, the reported performance metrics of these techniques are consistently high, with accuracy frequently exceeding 95% and F1 scores approaching 0.99, underscoring their potential for reliable real-time fall detection.

Machine learning has significantly advanced the development of fall detection systems, especially for elderly populations who are more vulnerable to falls. Various ML techniques, such as supervised learning, deep learning, and sensor fusion, have been applied to improve the accuracy and robustness of these systems (shown in Table 4). For instance, the authors of [34] employed supervised learning on synthetic datasets derived from IMU signals, establishing a controlled baseline for fall detection, while the authors of [35] utilized EEG data to capture postural changes, achieving 95.2% accuracy. This comparison illustrates how the choice of sensor data—IMU versus EEG—can affect detection outcomes, with each method catering to different aspects of human motion and physiological responses.

Deep learning frameworks have shown notable success in fall detection, with models such as those used by the authors of [36] incorporating 3D convolution and multilayer perceptron classifiers to achieve an F1 score of 0.9949. Additionally, the authors of [37] employed convolutional neural networks (CNNs) using data from dual IMUs, achieving 98.97% accuracy. These approaches highlight the power of deep learning in improving detection accuracy, particularly in controlled settings. Multi-modal approaches, such as combining accelerometers, gyroscopes, and video, are gaining traction, with the authors of [38] using self-attention CNNs to achieve 96.41% accuracy.

However, there are several challenges with these models. Many studies rely on controlled environments or small datasets, limiting the generalizability of the models. Moreover, there is limited integration of diverse sensor types, such as thermal or audio sensors, which could improve accuracy in real-world conditions. Additionally, complex deep learning models often require significant computational resources, limiting their deployment on edge devices.

To address these challenges, future research should focus on expanding datasets to include diverse populations and real-world scenarios, integrating multi-modal sensors for more comprehensive data, and optimizing ML models for edge computing to improve scalability. These improvements can make fall detection systems more reliable and efficient for everyday use.

**Table 4:** Summary of Machine Learning for Fall Detection.

Target Group	Reference	Main Ideas	Applied Method	Performance
Elderly People	[34]	Two synthetic datasets and a dataset of human falls were used, which incorporated inertial measurement unit signals.	Local Lipschitz Quotient Analysis and Machine Learning	-
Elderly People	[35]	Responses to postural perturbations were identified using EEG data.	Machine Learning and Sensor Fusion	95.2% accuracy, 90.9% sensitivity, 97.3% specificity
Elderly People	[39]	A supervised learning approach with a deep learning model was used to classify normal and fall scenarios. An unsupervised learning model was developed using an autoencoder model.	Threshold-Based Supervised Models Unsupervised Models	98.86% accuracy, F1 score of 0.99
Elderly People	[40]	The YOLO method was used for object detection and tracking in videos.	YOLO, Inception-v3, VGG-19	99.83% accuracy
Elderly People	[41]	A high-accuracy inertial sensor-based fall risk assessment tool was combined with machine learning algorithms.	Machine Learning	97.93% average accuracy
Elderly People	[42]	A preprocessing module, based on a mixed-radix FFT, was used for radar signal preprocessing. An NN accelerator was designed to support the updated block-wise (UBwise) computation technique. A fully connected (FC) layer cache compression technique was employed to reduce the cache required for FC layer computations.	Neural Network Machine Learning	98.58% accuracy
Elderly People	[43]	The work proposes a memory occupancy model for LSTM-type networks to pave the way for more efficient embedded implementations.	LSTM, STM8L Low-Power Processor	96.52% accuracy
Children, Healthy Adults, and Elderly People	[44]	The performance of three LSTM variants and two Transformer model variants for learning fall patterns was compared.	CNN-LSTM Model Transformer	-
Elderly People	[36]	A feature extractor built with a 3D convolution-based ResNet architecture was used to extract signal features, and a multilayer perceptron was employed as the classifier for fall detection.	3D Convolution Multilayer Perceptron	F1 score of 0.9949
Elderly People	[37]	The wearable device was equipped with dual IMUs on the waist and thigh, utilizing a CNN classification model, which was rigorously tested for its effectiveness.	CNN	98.974% accuracy
Elderly People	[45]	Artificial vision based on deep learning techniques was used.	CNN	94.8% accuracy, 93.1% sensitivity, 96.6% specificity
Children, Healthy Adults, and Elderly People	[38]	Based on triaxial acceleration and gyroscope data, a novel deep learning architecture—a Dual-Stream Convolutional Neural Network with Self-Attention (DSCS) model—was proposed.	DSCS, Deep Learning	96.41% accuracy, 95.12% sensitivity, 97.55% specificity
Elderly People	[46]	A comprehensive large-scale dataset was introduced, and a progressive enhancement of the YOLOv8s model was presented by integrating the Convolutional Block Attention Module (CBAM) at feasible stages of the network.	Enhanced Version of YOLOv8	-
Children, Healthy Adults, and Elderly People	[47]	The classifier is an ensemble of a CNN with residual connections and a bidirectional gated recurrent unit (BiGRU). The proposed model was evaluated using three different input dimensions: 6D input (including 3D acceleration and 3D angular velocity), 3D input (3D acceleration), and 1D input (the magnitude of 3D acceleration).	CNN	F1 score of 0.98
Children, Healthy Adults, and Elderly People	[48]	Through the design and experimental validation of a radar-based fall detection system, effective technical support was provided for monitoring falls in elderly individuals using spectral and deep learning methods.	CNN, Bidirectional Long Short-Term Memory	98.83% accuracy
Elderly People	[49]	Edge computing was used for data preprocessing, while the classification process, based on multistream hierarchical learning, was performed in the cloud.	Multistream Hierarchical Learning	average F1 score of 0.9781
-	[50]	The raw data from depth images were used to establish a ground reference to improve fall detection accuracy, while a background subtraction algorithm was applied for comprehensive analysis to distinguish foreground elements.	SVM, Multilayer Perceptron, Radial Basis Function, Neural Network (NN)	95% accuracy
Children, Healthy Adults, and Elderly People	[51]	A fine-tuned AlphaPose was used to extract 2D human skeleton sequences from infrared videos. The skeleton data, represented in both Cartesian and polar coordinates, were processed by a dual-stream ST-GCN for rapid fall behavior recognition.	Spatial–Temporal Graph Convolutional Network	96% accuracy
Patients and Elderly People	[52]	A hybrid Parallel Convolutional Neural Network and Transformer-based architecture (PCNN–Transformer) was used.	Parallel Convolutional Neural Network (PCNN), Transformer	average accuracy of 99.45%
Elderly People	[53]	CNN-LSTM, RNN-LSTM, and GRU-LSTM models were used, with and without attention layers, and performance metrics were analyzed to identify the optimal deep learning model. Additionally, three different hardware boards (the Jetson Nano development board, Raspberry Pi 3, and Raspberry Pi 4) were used as AI edge computing devices.	CNN, RNN, LSTM, GRU	97% accuracy, 98% sensitivity, 98% specificity, F1 score of 0.98
Elderly People	[54]	By learning fast and slow falls separately, the model could identify various types of falls. A “Timer LSTM” was developed, and data ambiguity was further analyzed using the Lipschitz quotient as a measure of data deficiency.	LSTM	97% accuracy
Elderly People	[55]	A novel Transformer-based model was employed.	Transformer	-
Children, Healthy Adults, and Elderly People	[56]	With an additional set of 10 videos specifically added for testing purposes, the RetinexNet algorithm was used to preprocess the acquired images. The YOLOv5 network was improved by integrating the CBAM and TPH modules, along with a comprehensive augmentation strategy, enhancing the network’s ability to capture and extract fall detection features.	YOLOv5	96.52% accuracy
Children, Healthy Adults, and Elderly People	[57]	An unsupervised RGB-to-Depth (RGB2Depth) cross-modal domain adaptation method was proposed.	Deep Unsupervised RGB2Depth Adaptation	-
Elderly People and People with Disabilities	[58]	This framework employed edge computing for efficient analysis and used the MHEALTH dataset.	Machine Learning	96% accuracy, F1 score of 0.9052,
Elderly People	[59]	RFID tags were integrated with an RFID reader into a smart carpet, where the embedded RFID tags transmit signals to the RFID reader, effectively distinguishing fall events from normal movements.	KNN	99.97% accuracy, 0.999 sensitivity, 0.999 specificity, F1 score of 0.999
Patients with Parkinson’s Disease	[60]	FoG-Net consists of a backbone network and a feature fusion network, where the backbone network extracts shallow temporal features, and the feature fusion network utilizes a self-attention mechanism to automatically learn intra-token information.	Deep Neural Network	96.97% accuracy
Elderly People	[61]	A lightweight convolutional neural network extracted motion features from multi-modal data for activity recognition. A decision fusion algorithm combined the fuzzy comprehensive evaluation method and majority voting strategy to determine the weight values influencing the final decision outcome.	LCNN	95.31% accuracy

## 4. Discussion

This bibliometric analysis revealed key research hotspots and collaboration networks in wearable sensor-based fall detection over the past decade. Notably, the growing volume of publications and the extensive international collaborations underscore the global interest in integrating advanced computational methods—such as machine learning and deep learning—with wearable sensor technologies. Analyses of keyword co-occurrences and network maps indicate emerging research clusters focused on optimizing sensor performance, enhancing data fusion techniques, and developing predictive models for fall detection. These findings provide valuable insights into current research trends and highlight the importance of interdisciplinary approaches to advance the design and utilization of robust fall detection systems in real-world settings.

The results of this study reveal a marked increase in publications related to wearable sensors for fall detection, reflecting the rising attention the academic community is giving to this field [62,63,64]. As interest from academia and educational institutions has continued to grow, the volume of publications on wearable sensors for fall detection has steadily increased over the years [65]. This trend highlights the increasing recognition of the potential of wearable devices to improve fall detection systems.

Furthermore, the analysis emphasizes the strength of international collaboration, with research efforts concentrated in both developed and developing countries/regions. This pattern demonstrates the global interest in leveraging wearable technologies for fall detection and prediction. Keywords such as “wearable sensors”, “fall detection”, “fall prevention”, and “machine learning” have emerged as key nodes in the co-occurrence network, reflecting evolving research priorities and interdisciplinary connections across various domains.

Progress Made in Wearable Fall Detection Systems

The integration of advanced sensor technologies, such as triaxial accelerometers, gyroscopes, and pressure sensors, has greatly improved the accuracy and reliability of wearable devices for fall detection. These technologies, combined with machine learning and deep learning algorithms, have increased the precision of detecting falls while minimizing false alarms [52,66]. Additionally, there has been significant progress in real-time, continuous monitoring, allowing for the early detection of fall risks and preventing incidents before they occur.

Another noteworthy advancement is the incorporation of the Internet of Things (IoT) and Artificial Intelligence of Things (AIoT) into wearable fall detection systems. These innovations have paved the way for interconnected devices that not only detect falls but also trigger immediate alerts to caregivers or healthcare providers, enabling faster response times. The focus on personalizing fall detection systems, considering factors such as health conditions, mobility, and environmental conditions, has enhanced the adaptability and effectiveness of these devices [67].

Moreover, the increasing collaboration between worldwide institutions has accelerated the development of these technologies, with institutions in China, the United States, South Korea, and India playing pivotal roles in pushing the boundaries of wearable fall detection systems.

Future Research Directions

Despite the progress made, several aspects of these models can benefit from further advancement. One promising direction is the continued integration of wearable sensors with the IoT and AIoT. Future research should focus on improving the interoperability between these devices and IoT networks, ensuring seamless data transfer, and refining AI models to provide more personalized fall detection systems. Such advancements could lead to the development of highly adaptive systems that cater to individual health profiles, activity levels, and environments.

Another area for exploration is the development of more sophisticated predictive algorithms. While current systems are effective at detecting falls, future advancements should focus on predicting falls before they happen [68]. Machine learning models that analyze gait patterns, movement abnormalities, or environmental factors could help anticipate a fall and take preventive action, such as alerting the user or initiating safety measures.

Limitations in the comfort and energy efficiency of wearable sensors should also be addressed. Future work should focus on optimizing the design of these devices to ensure that they are comfortable for long-term use, especially for elderly individuals, while improving battery life to accommodate continuous, real-time monitoring without frequent recharging.

Furthermore, a more user-friendly design for elderly users is a crucial aspect that should be integrated into future fall detection systems. This involves designing devices that are ergonomically optimized with intuitive interfaces, lightweight materials, and unobtrusive form factors [69,70]. By emphasizing these types of designs, these systems can become more accessible and appealing to the elderly, ultimately encouraging wider adoption.

Additionally, research into the standardization of wearable fall detection systems is crucial. Developing universal testing protocols and performance metrics will ensure that fall detection devices meet the necessary safety and reliability standards for wide-scale deployment.

Challenges to Overcome

Several challenges remain for wearable fall detection systems to reach their full potential. One major issue is ensuring data privacy and security, as these devices collect sensitive personal health data. Future research should prioritize the development of secure data transmission and storage methods, ensuring that user privacy is protected.

Cost is another significant barrier, particularly in low-income regions where access to healthcare technologies is limited. There is a need to make wearable fall detection systems more affordable without compromising their functionality. Additionally, ensuring that these devices are easy to use and do not cause discomfort or disruption to daily life is critical for widespread adoption among elderly users.

Conclusions and Future Outlooks

This study contributes to the field by systematically surveying current review papers on fall-related systems, an aspect that has been rarely addressed in previous research [15]. Moving forward, we aim to expand our research to incorporate additional studies and explore further advancements in the integration of wearable sensors with other technologies for fall detection and prevention. By addressing the challenges of cost, comfort, and privacy, as well as enhancing predictive capabilities and system integration, wearable sensors for fall detection are expected to play a pivotal role in improving elderly care and reducing the incidence of fall-related injuries worldwide.

## Figures and Tables

**Table 1:** Top 10 productive countries/regions.

Country/Region	Count	Centrality
China	154	0.26
U.S.A	95	0.31
India	48	0.12
South Korea	48	0.04
Spain	45	0.12
Italy	44	0.04
England	39	0.18
Taiwan, China	35	0.01
Australia	34	0.06

**Table 2:** Top 10 keywords.

Keywords	Count	Centrality
Fall Detection	358	0.03
System	113	0.01
Wearable Sensors	87	0.05
Machine Learning	77	0.01
Deep Learning	68	0.03
Older Adults	65	0.03
People	62	0.02
Activity Recognition	59	0.09
Classification	53	0.06
Recognition	51	0.01
